# The Effect of Tobacco Control Mass Media Campaigns on Smoking-Related Behavior Among People With Mental Illness: A Systematic Literature Review

**DOI:** 10.1093/ntr/ntac079

**Published:** 2022-03-31

**Authors:** Parvati R Perman-Howe, Ann McNeill, Leonie S Brose, Bernadett E Tildy, Tessa E Langley, Debbie Robson

**Affiliations:** Addictions Department, King’s College London, London, UK; Addictions Department, King’s College London, London, UK; Addictions Department, King’s College London, London, UK; Addictions Department, King’s College London, London, UK; University of Nottingham, School of Medicine, Nottingham, UK; Addictions Department, King’s College London, London, UK

## Abstract

**Introduction:**

Tobacco control mass media campaigns (MMCs) can be effective generally, but little is known about their effects among people with mental illness. The objectives of this study were to systematically review: (1) Whether tobacco control MMCs affect smoking-related outcomes among people with mental illness. (2) Cost-effectiveness.

**Aims and Methods:**

Data sources: MEDLINE, Embase, PsycInfo, Web of Science, CINAHL, the Cochrane Library (searched March 2021), reference lists of included articles and relevant systematic reviews. Study eligibility criteria: Population: Adults with mental illness and experience of smoking tobacco and/or using other nicotine-containing products. Intervention/exposure: Tobacco control MMC messages. Comparator: No exposure, other tobacco control intervention(s), no comparator. Primary outcome: Changes in quitting behaviors. Study design: All primary research. Quantitative data were appraised using the EPHPP tool, qualitative data using CASP’s Studies Checklist. Data were synthesized narratively.

**Results:**

Eight studies were included, seven were at high risk of bias. There was inconclusive evidence of the effect of MMCs on quit attempts and intentions to quit among people with mental illness. Increasing advertisement exposure did not increase quit attempts or intentions to quit among those with mental illness, however, increased exposure to an advertisement that addressed smoking and mental health did. None of the studies assessed cost-effectiveness.

**Conclusions:**

Findings should be interpreted with caution as data are limited and of low or moderate quality. There is evidence to suggest that tobacco control MMCs have limited impact on those with mental illness, although campaigns that are specific to smoking and mental health may be effective.

**Implications:**

There is a paucity of good-quality evidence of the effect of tobacco control MMC messages among people with mental illness. Careful consideration should be given to the design of future studies that evaluate MMCs in order to minimize the risk of bias, establish causality, and ensure the findings reflect real-world implementation. Further research should examine the need for MMC messages that address mental health.

## Introduction

### Background

Tobacco smoking (hereafter referred to as smoking) is one of the main causes of preventable death, disease, and disability worldwide.^1^ In 2019, approximately 14.1% of adults in the United Kingdom smoked tobacco cigarettes.^2^ Smoking prevalence is around 50% higher among people with common mental health conditions such as depression, generalized anxiety disorder (GAD), and obsessive-compulsive disorder (OCD).^3^ It is almost three times higher among those with serious mental illness (SMI) that includes schizophrenia, bipolar disorder, or other psychoses.^4^ Furthermore, people who experience mental illness are more likely to be heavy smokers,^3,5^ and experience harm from smoking,^5,6^ than people without mental illness. These disparities are also present in other industrialized countries, for example, the United States.^6–8^

The mass media is defined as channels of communication (such as television, radio, online broadcasting, social media, email, websites, mobile apps, newspapers, magazines, leaflets, booklets, billboards, or posters) intended to reach large numbers of people, and which are not dependent on person-to-person contact.^9^ Public health messages are often incorporated in mass media campaigns (MMCs), which aim to influence health behaviors and the individual-level determinants of health behaviors.^10^ Individual-level determinants are factors that can influence behavior such as awareness, knowledge, beliefs, attitude, norms, self-efficacy, motivation, and intentions.^10^ Tobacco control MMCs are intended to decrease tobacco consumption and smoking prevalence and increase quitting-related behaviors such as quit attempts and the use of smoking cessation products.^11^ MMCs may be a cost-effective way to reduce tobacco use on their own^10^ or as part of a wider tobacco control programme.^12–14^ Their effectiveness depends on the content, tone, and style of the health messages and the level of individual exposure.^13–16^ Messages that are emotive,^10^ that convey the negative health effects of tobacco,^10,13^ and that denormalize smoking behavior^10^ may be the most effective although the evidence is inconsistent.^17^ Evidence suggests that, at the population level, tobacco control MMCs can have a positive effect on attitudes and intentions^10,13^; can increase quit attempts,^13^ abstinence,^14^ and calls to smoking quitlines^10^; and can reduce smoking prevalence.^12–14^ However, little is known about their effectiveness among people who experience mental illness, and this is yet to reviewed systematically.

### Review Question

What is the effect of tobacco control MMCs on quitting, smoking-related behavior and individual-level determinants among people with mental illness?

### Objectives

1. To assess whether tobacco control MMCs result in a change in the following outcomes among people with mental illness:
  - Quitting behaviors (quit attempts and cessation).
  - Combustible tobacco consumption.
  - Smoking prevalence.
  - Treatment-seeking behavior.
  - Use of smoking cessation products.
  - Use of e-cigarettes.
  - Individual-level determinants of smoking-related behavior.
2. To assess the cost-effectiveness of tobacco control MMCs among people with mental illness.

## Methods

### Protocol and Registration

The review protocol was registered in PROSPERO on February 11, 2021. Registration number: CRD42021236066. The protocol and supporting documents are published on the Open Science Framework (OSF) website: https://osf.io/gda3y/. The review adheres to the Preferred Reporting Items for Systematic Reviews and Meta-Analyses (PRISMA) statement.^18^

### Eligibility Criteria

#### Study Design

Primary research: not restricted to specific study designs and included studies that elicited quantitative and/or qualitative data.

#### Population

To be included, studies had to include people who:

- were aged 18 years or older and
- have, or have had, a diagnosis of one or more mental health condition(s) and/or
- screened positive for mental distress and/or one or more mental health condition(s) and
- have experience of smoking tobacco and/or using other nicotine-containing products.

#### Intervention/Exposure

MMC tobacco control public health messages. The MMC could be either carried out alone or in conjunction with other tobacco control programmes provided the contribution and effectiveness of the mass media component could be assessed.

#### Comparator

The comparator depended on the study design and included: no exposure to the MMC, for example, as a nonexposed control in a randomized controlled trial (RCT) or in the before phase of a before and after study, no comparator, for example, in a cross-sectional study, and other tobacco control intervention(s), for example, taxation in a natural experiment.

#### Primary Outcome

Changes in quitting behaviors (quit attempts and cessation). A quit attempt was defined as an attempt made to discontinue smoking that lasted for any period of time. Cessation was defined as the complete discontinuance of smoking. Measures included self-reported and/or biochemically validated smoking status (e.g., expired breath carbon monoxide levels). The measurements could be made at different times depending on the study design.

#### Additional Outcomes

Additional outcomes included but were not limited to: changes in combustible tobacco consumption; changes in smoking prevalence; changes in treatment-seeking behavior (e.g., calls to telephone quitlines); changes in use of smoking cessation products (nicotine replacement therapy [NRT]); changes in use of e-cigarettes; recall; changes in individual-level determinants of smoking-related behavior including awareness, knowledge, beliefs, attitude, norms, self-efficacy, motivation and/or intentions; cost-effectiveness of campaign. Measures included self-reported consumption; sales data (e.g., NRT and e-cigarette sales), telephone call data (e.g., from quitlines), cost-effectiveness data (e.g., cost per life-year saved). The measurements could be made at different times depending on the study design. For example, an interrupted time series may measure the number of calls to quitlines before and after the launch of the campaign, whereas a randomized controlled trial may measure the number of calls to quitlines after randomization.

### Search Strategy

MEDLINE, Embase, PsycInfo, Web of Science, CINAHL, and the Cochrane Library were searched between March 30 and 31, 2021. Supplementary Material S1 details the full search strategy.

### Study Selection

**Figure 1.**
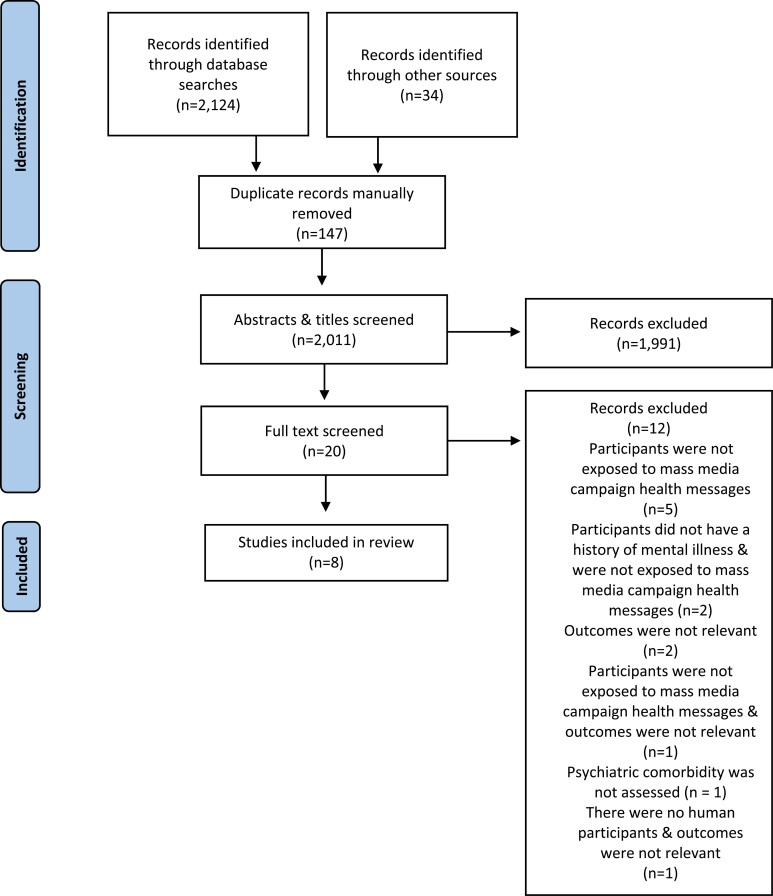
The study selection process.

Articles were exported into Endnote and Cadima where duplicates were removed by hand.^19^ Two reviewers (PPH and BT) performed a consistency check by screening the titles and abstracts of 10% of the articles. Results indicated a “fair” inter-rater agreement (Kappa value: 0.57; inconsistencies in 41/198 articles). The two reviewers discussed the inconsistencies and came to a consensus as to which articles were eligible. They also discussed and amended the screening checklist. The two reviewers screened another 50 titles and abstracts using the amended checklist. This produced no inconsistencies (Kappa value: 1). PPH then screened the titles and abstracts of the remaining articles. Following this, both reviewers independently screened the full text of 10% of articles. Again, the two reviewers discussed the inconsistencies and came to a consensus as to which articles were eligible. PPH then screened the full text of the remaining articles. The reference lists of included articles and relevant systematic reviews were used to identify other primary research studies. Figure 1 illustrates the study selection process.

### Data Collection

Data from included studies were extracted into a pre-piloted form (Supplementary Material S2). Extracted data were independently checked by a second reviewer (DR) for accuracy and completeness. Where necessary, authors were contacted to provide additional information. Where data were not provided, they were reported as “missing.”

### Risk of Bias

The Effective Public Health Practice Project (EPHPP) quality assessment tool was used to appraise studies that provided quantitative data.^20–22^ The EPHPP tool provides a global rating based on six main elements: selection bias, study design, confounders, blinding, data collection methods, and withdrawals and dropouts. The Critical Appraisals Skills Programme’s (CASP’s) Qualitative Studies Checklist was used to appraise qualitative data.^23^ The CASP Checklist assesses nine main features: statement of aims, appropriate methodology, appropriate design, appropriate recruitment strategy, appropriate data collection, consideration of the researcher and participant relationship, consideration of ethical issues, rigorous data analysis, and statement of findings. Two reviewers (PPH and BT) independently assessed the quality of included studies, discussed any inconsistencies and came to a consensus for the overall rating.

### Synthesis Methods

Due to heterogeneity in study design and outcomes, a narrative synthesis was used.

## Results

### Study Selection

The search strategy identified 2011 unique articles, of which 8 were included in the narrative synthesis (Figure 1). Reasons for excluding each article based on the full-text screen are listed in Supplementary Material S7.

### Study Characteristics

Table 1 shows the characteristics of the eight included studies. Studies were conducted in the USA (*n* = 6)^24–29^ and Australia (*n* = 2).^30,31^ Seven of the studies used quantitative methods^24–30^ and one used mixed methods.^31^ Study designs included cross-sectional,^25,29–31^ before and after (pre-post)^24,26,27^ and a cluster randomized field experiment.^28^ Sample sizes ranged from 89^31^ to 22 965^25^ for the quantitative components. Eight people took part in a semistructured telephone interview as the qualitative component of the mixed-methods study.^31^

**Table 1.** Characteristics of Included Studies Organized by Reported Outcomes

Author, year, country	Campaign name (independent variable of interest) and dates	Study design	Sample size (with mental illness)	Mental health definition/measure	Outcomes and outcome measures
Neff et al., 2016, USA^26^	Tips campaign, 7 July–7 Sept 2014	Before and after	4248 (1388)	Not stated	Change in quit attempts (past 3 months): OR Change in intentions to quit (next 30 days): OR Change in intentions to quit (next 6 months): OR
Davis et al., 2017, USA^24^	Tips campaign (PE), 3 Feb–6 April and 10 July–7 Sept 2014	Before and after	7217 (2214)	Self-report of any diagnosis of depression, anxiety disorder, ADHD/ADD, or other mental health condition	Quit attempts (past 3 months): OR Perceived campaign effectiveness: B-coef
Davis et al., 2018, USA^25^	Tips campaign (exposure), 2012–2015	Cross-sectional	22 965 (6761)	Self-report of any diagnosis of depression, anxiety disorder, general mental health conditions, ADHD/ADD	Quit attempts (past 3 months): AOR Intentions to quit (next 30 days): AOR
Prochaska et al., 2018, USA^27^	Tips campaign (exposure), 25 Jan–12 June 2016	Before and after	2583 (777)	Self-report of any diagnosis of depression, anxiety disorder, ADHD/ADD, or other mental health condition	Quit attempts (past 6 months): Freq, %, AOR Intentions to quit (next 30 days): Freq, %, AOR Ad exposure (based on ad recall): %
McAfee et al., 2017, USA^28^	Tips campaign (exposure), 4 March–21 June 2013	Randomized field experiment	8576 (2533)	Not stated	Quit attempts (since campaign launch—past 6 months 27 days): %, AOR
Nonnemaker et al., 2014, USA^29^	New York State’s antismoking advertising (exposure), no dates for campaign provided(data were collected 2003–2011)	Cross-sectional	9408 (missing data)	Self-report: “Now thinking about your mental health, which includes stress, depression, and problems with emotions, for how many days during the past 30 days was your mental health not good?’ Poor mental health was defined by responses of 10 or more days, to ensure that at least 10% of the sample would be defined as having poor mental health	Quit attempts (past 12 months): Freq, %, OR
Thornton et al., 2013, Australia^30^	Australia’s tobacco, alcohol and cannabis public health campaigns (exposure and PE), no dates provided	Cross-sectional	1046 (137)	All participants recruited via the Australian Schizophrenia Research Bank (ASRB) had a diagnosed psychotic disorder. Additionally, self-report of a diagnosis of a mental disorder (depression, anxiety, eating disorder, psychotic disorder or other). Current psychological distress was measured using the Depression, Anxiety and Stress Scale (DASS-21).	Perceived campaign effectiveness: % Ad exposure (based on ad recall): %
Thornton et al., 2011, Australia^31^	Australia’s anti-smoking public health campaigns (exposure), no dates provided	Cross-sectional (using mixed methods)	Quantitative component: 89(89) Qualitative component: 8(8)	Participants were registrants of the ASRB, which contains data about people with a clinical diagnosis of schizophrenia and related disorders	Ad exposure (based on ad recall; quantitative): Freq, % Perceived campaign effectiveness (qualitative) Knowledge (qualitative)

ADHD/ADD = attention-deficit hyperactivity disorder/attention-deficit disorder; Ad(s) = advertisement(s); AOR = adjusted odds ratio; B-coef = beta coefficient; Freq = frequencies; OR = odds ratio; PE = perceived effectiveness; Tips = Tips From Former Smokers campaign.

Five of the studies assessed the Tips From Former Smokers (Tips) campaign.^24–28^ The Tips campaign was launched in 2012 by the Centers for Disease Control and Prevention (CDC) in the United States with the aim of encouraging people to quit smoking. The primary audience is adult smokers aged 18–54. The campaign consists of television advertisements supported by complementary advertisements on the radio, online, print and billboards. Tips campaign advertisements feature graphic, emotional messages from people living with serious long-term health conditions as a result of smoking and/or secondhand smoke exposure. The latest campaign wave was launched in March 2021.^32^ Of the remaining three studies, one assessed New York State’s smoking cessation advertisements from 2003 to 2011.^29^ These advertisements incorporated graphic images, emotional content, and information on quitting and the dangers of secondhand smoke. The remaining two studies assessed historical tobacco control public health campaigns in general in Australia but no further details about the campaigns, including their names, were provided.^30,31^ Only one study assessed a campaign which included specific advertisements that targeted people with mental illness.^27^ One study looked at the effect of MMCs on people with mental illness only.^31^ Outcomes reported were: quit attempts (*n* = 6 studies),^24–29^ intentions to quit (*n* = 3 studies),^25–27^ advertisement exposure (measured as advertisement recall; *n* = 3 studies),^27,30,31^ perceived effectiveness (*n* = 3 studies),^24,30,31^ and knowledge of the health impact of smoking (*n* = 1 study).^31^ The term “quit attempts” was defined as “at least one quit attempt lasting at least 24-hours in the past X (number of) months” in all six studies that reported quit attempts as an outcome. None of the included articles reported smoking cessation, combustible tobacco consumption, smoking prevalence, treatment-seeking behavior, use of smoking cessations products, or use of e-cigarettes among people with mental illness as an outcome. Campaign cost effectiveness was also not reported as an outcome.

### Risk of Bias Within Studies

Seven studies had a high risk of bias and one had a moderate risk of bias as measured by the EPHPP tool^25,27–31^ (Supplementary Materials S3 and S4). The most common sources of bias were lack of blinding (in all eight studies), study design (four studies were cross-sectional), and data collection methods (four studies did not demonstrate that data collection tools were valid and reliable). The qualitative component of the mixed methods study had a low risk of bias across five elements of the CASP Checklist: statement of aims, appropriate methodology, appropriate design, rigorous data analysis, and statement of findings^31^ (Supplementary Materials S5 and S6). However, it did not include sufficient information to assess risk of bias for four elements: appropriate recruitment strategy, appropriate data collection, consideration of the researcher and participant relationship, and consideration of ethical issues.

Table 2 and Supplementary Material S8 show the key results from each of the eight included studies ordered by reported outcomes and length of follow-up.

**Table 2.** Qualitative Results From the Mixed-Methods Study

Author, year	Outcome	Theme	Subtheme	Illustrative quotes
Thornton et al., 2011**^31^**	Perceived effectiveness	Ineffectiveness of anti-smoking campaigns	1.Limited impact of antismoking campaigns	“There are very strong in your face ads . . . they don’t get you to give up cigarettes as strongly as I think the ads might like to think”
			2.Disregard of antismoking messages	“People with mental illness . . . they’d pay no attention to them I wouldn’t think, unless they’re real paranoid”
			3.Relationship between motivation and campaign effectiveness	“. . . an ad’ll come on and triggers cigarette thoughts and . . . it would make you want to have a cigarette . . . but towards the end when I was getting in my head that I did want to give up then those ads . . . well, you watch them and you go ‘Oh I got to give up’”
	Knowledge	Health impacts of tobacco	1.Tobacco’s negative physical health impact	“Cigarettes would be the most physically harmful . . . like what cigarettes do to you and how harmful they are to the lungs and the mouth and all parts of the body”
			2.Tobacco’s positive mental health impact	“It’s a high addictive antidepressant and you know it works better than an antidepressant cos it gives you that instant effect that you’ve had some sort of mood relaxant . . . a bit of a stress relief against depression”

## Quitting Behaviors

### Quit Attempts

Six studies reported quit attempts as an outcome.^24–29^ One was classified as having a moderate risk of bias^26^ and five were classified as having a high risk of bias^24,25,27–29^ (Supplementary Material S8).

Neff et al. looked at the change in quit attempts at 3 months after (compared to before) the Tips campaign.^26^ This was the one study that was classified as having a moderate risk of bias. It found no changes in quit attempts following the campaign (compared with before the campaign) for those with mental illness, whereas those without mental illness were more likely to have made a quit attempt following the campaign^26^ (Supplementary Material S8).

Davis et al. (2017) looked at the effect of perceived effectiveness of the campaign on quit attempts at 3 months.^24^ There was no difference in quit attempts for those with, compared with without, mental illness^24^ (Supplementary Material S8).

Three studies reported the proportion of participants with and without mental illness who made a quit attempt at either 6^27,28^ or 12^29^ months following a tobacco control campaign. This was reported either at follow-up in a before and after study^27^ and a randomized field experiment,^28^ or in a cross-sectional study.^29^ This ranged from 39.5%^28^ to 57.7%^29^ among those with mental illness and from 32.0%^28^ to 53.1%^29^ among those without. In general, all three studies found that a larger proportion of those with mental illness had made a quit attempt than those without. This was significant in one study.^27^ In another study, this was significant with a standard level of exposure (761 national gross rating points [GRPs: a measure of exposure based on the proportion of the population who are potentially exposed to advertisements and the average number of times the advertisements may have been seen over a time period]), but level of significance was not reported for a higher level of exposure (758 national GRPs plus 1724 local GRPs) although the percentages reporting quit attempts were similar (39.5% with and 38.5% without a mental health condition).^28^ In the third study, level of significance was not reported^29^ (Supplementary Material S8).

Four studies focused on the effect of differential levels of advertisement exposure on quit attempts at 3,^25^ 6,^27,28^ and 12 months.^29^ For generic advertisements, Davis et al. (2018) reported data for quit attempts for the overall sample but they did not report associations by mental health status.^25^ The authors stated that there were no significant interactions between GRPs and mental health status for quit attempts. However, it cannot be assumed that the associations were the same for those with and without mental illness. Therefore, the reported significant association between GRPs and quit attempts should be regarded with caution when only considering the effect of advertisement exposure on quit attempts among those with mental illness. Prochaska et al. found that those with mental illness were not more likely to make a quit attempt with self-reported increased frequency of exposure to generic campaign advertisements.^27^ The opposite was found for those without mental illness.^27^ McAfee et al. found that increased (758 national GRPs plus 1724 local GRPs), compared to standard (761 national GRPs), exposure to advertisements did not increase the likelihood of those with mental illness making a quit attempt, while those without mental illness were more likely to make a quit attempt with increased, compared to standard, exposure.^28^ Nonnemaker et al. found that among those with mental illness the following had no impact on quit attempts: advertisement recall, recall of graphic/emotional advertisements, greater GRPs, greater GRPs for graphic/emotional advertisements, or greater GRPs for a comparison advertisement.^29^ In contrast, those without mental illness were more likely to make a quit attempt with advertisement recall, recall of graphic/emotional advertisements, greater GRPs and greater GRPs for graphic/emotional advertisements.^29^ They were not more likely to make a quit attempt with greater GRPs for a comparison advertisement (Supplementary Material S8).

The only study that focused on mental health specific advertisements found that those with mental illness were more likely to make a quit attempt with self-reported increased frequency of exposure to an advertisement that was specific to smoking and mental health.^27^ The opposite was found for those without mental illness^27^ (Supplementary Material S8).

Combined, findings from these studies suggest that increasing exposure to tobacco control campaign advertisements did not increase quit attempts among those with mental illness.^27–29^ The one exception appears to be that increasing exposure to advertisements that focus on smoking and mental health increased quit attempts among those with mental illness.^27^

## Self-reported Advertisement Recall/Advertisement Exposure

Three studies used self-reported advertisement recall as a proxy measure for advertisement exposure.^27,30,31^ These studies only provided descriptive statistics. All three studies were classified as having a high risk of bias (Supplementary Material S8).

Prochaska et al.^27^ and Thornton et al. (2013)^30^ reported the proportion of participants with and without mental illness who saw at least one tobacco control campaign/advertisement. The differences were non-significant in both studies. Prochaska et al. also reported that there was no significant difference in the proportion of those with and without mental illness who reported seeing an advertisement that focused on smoking and mental health or at least one non-mental health specific campaign advertisement.^27^ Thornton et al. (2011) only included participants with a psychotic disorder.^31^ Of 88 participants, 82 (93.2%) reported having been exposed to at least one tobacco control campaign. These figures were similarly as high in the other study by Thornton et al., in which the population with mental illness also had a primary diagnosis of a psychotic disorder.^30^ In contrast, the figure was lower in the Prochaska study that measured mental health by self-report of lifetime diagnosis of any mental health condition^27^ (Supplementary Material S8).

## Individual-Level Determinants of Smoking-Related Behavior

### Intentions to Quit

Three studies reported intentions to quit as an outcome.^25–27^ One was classified as having a moderate risk of bias^26^ and two were classified as having a high risk of bias^25,27^ (Supplementary Material S8).

Neff et al. looked at the change in intentions to quit in the next 30 days and the next 6 months before and after the Tips campaign.^26^ They found no changes following the campaign (compared with before the campaign) in reported intentions to quit among those with mental illness within the next 30 days or the next 6 months. There were also no changes in reported intentions to quit within the next 30 days among those without mental illness. However, those without mental illness had an increased likelihood of reporting intentions to quit within the next 6 months following the campaign (Supplementary Material S8).

Two studies looked at the effect of advertisement exposure on intentions to quit within the next 30 days.^25,27^ For generic advertisements, the data between the two studies that looked at the effect of advertisement exposure on intentions to quit are not comparable because one of the studies only reported data for the overall sample rather than by mental health status. The authors of this article stated that there were no significant interactions between GRPs and mental health status for intentions to quit.^25^ Prochaska et al. described the proportion of participants with and without mental illness who reported intentions to quit following a tobacco control campaign.^27^ No difference between the two groups was detected. The authors also found that both those with and without mental illness were not more likely to report intentions to quit with self-reported increased frequency of exposure to generic campaign advertisements (Supplementary Material S8).

The only study that focused on mental health specific advertisements found that those with mental illness were more likely to report intentions to quit with self-reported increased frequency of exposure to an advertisement that was specific to smoking and mental health.^27^ The opposite was found among those without mental illness (Supplementary Material S8).

### Perceived Effectiveness

Three studies with high risk of bias reported perceived effectiveness as an outcome^24,30,31^ (Supplementary Material S8).

Davis et al. (2017) reported that those with mental illness reported a higher level of perceived effectiveness than those without.^24^ Similarly, Thornton et al. (2013) found that a larger proportion of participants with mental illness said they perceived tobacco control campaigns to be effective compared to those without mental illness^30^ (Supplementary Material S8). However, qualitative data from Thornton et al. (2011) suggest that those with mental-ill health tended to perceive tobacco control campaigns as ineffective.^31^ This perception was characterized by three themes: the limited impact of antismoking campaigns, the disregard of antismoking messages, and the relationship between motivation and campaign effectiveness (Table 2). Given details of the campaigns were not available for Thornton studies, these findings cannot be explored further.

### Knowledge

One study with high risk of bias reported knowledge as an outcome^31^ (Supplementary Material S8).

Qualitative data from Thornton et al. (2011) suggest that tobacco control MMCs can increase knowledge of the negative physical health impacts of tobacco use, among those with mental illness.^31^ However, findings also suggest that among this population there are perceptions that tobacco use has a positive impact on mental health (Table 2).

## Discussion

Overall, there is inconclusive evidence of the effect of tobacco control MMCs on quit attempts and intentions to quit among people with mental illness. Advertisement exposure was generally high, and similar, among those with and without mental illness. In general, increasing advertisement exposure did not increase quit attempts or intentions to quit among those with mental illness. However, in the one study that assessed a campaign tailored to mental health, increased exposure to an advertisement that addressed smoking and mental health did increase both quit attempts and intentions to quit among this population. Findings should be regarded with caution as data were derived from only eight studies, seven of which were at high risk of bias.

### Quit Attempts

Findings from this review on the effect of tobacco control MMCs on quit attempts among those with mental illness are inconclusive. No existing reviews have looked at this. A review of reviews found two studies that examined the effect of tobacco control MMCs on general populations.^10^ Of these, one reported mixed results whilst the other reported no effect. However, the latter review had a high risk of bias and evidence was derived from only one study. A Cochrane review found that state-wide and community-based tobacco control MMCs resulted in no significant changes, or differences between the control and intervention groups, in quit attempts among adults.^14^ However, this review did not focus on those with mental illness, the included studies were heterogenous and they tended to have a high risk of bias.^14^ Other studies have suggested that tobacco control MMCs motivate quit attempts, but their effectiveness depends on the content, tone and style of the health messages and the level of individual exposure.^10,13^ However, none of these studies focused on people with mental illness.

Findings for the effect of advertisement exposure on quit attempts suggest that increasing exposure to tobacco control campaign advertisements did not increase quit attempts among those with mental illness.^27–29^ The one exception appears to be that increasing exposure to an advertisement that focused on smoking and mental health increased quit attempts among those with mental illness.^27^ This advertisement, known as the “Rebecca” advertisement, featured testimonies from a 57-year-old woman with depression who had successfully quit smoking. There were notable similarities between the three studies that assessed the effect of advertisement exposure on quit attempts. They were all based in the United States, although one of the studies was restricted to New York State.^29^ Two studies assessed the Tips campaign,^27,28^ while the other assessed New York State’s smoking cessation campaigns.^29^ Furthermore, all three studies used a probability sample and a standardized definition of smoking. Nonetheless, the findings should still be interpreted with caution. Data were derived from only three studies, all of which had a high risk of bias. Mental illness was measured in different ways, which may suggest that the study populations were not comparable. Furthermore, increased exposure was operationalized differently between studies meaning that the data were not wholly comparable.

### Self-reported Advertisement Recall/Advertisement Exposure

Findings from this review suggest that those with SMI are more likely to remember being exposed to tobacco control advertisements than those reporting any level of mental illness but whether this is due to greater exposure is unknown. The difference in findings could be explained by the campaigns that were evaluated and the measures used to assess campaign exposure. The narrow focus of the Prochaska study^27^ on one iteration of one campaign over a relatively short length of time compared to two studies that asked if participants could recall ever having come across any campaigns^30,31^ may explain differences in reported exposure. Either way, findings should be interpreted with caution as data were derived from only three studies and all had a high risk of bias. Previous research has suggested that, among general populations, exposure to tobacco control MMCs is a key factor in enabling smoking-related behavior change.^15,16,33^ Langley et al. found that an increase in exposure to tobacco control advertisements was associated with an increase in calls to a stop smoking helpline in the same month in England and Wales.^16^ However, in subsequent months these findings did not reach significance. Another study found that average exposure to a tobacco control MMC of four times per month (390 GRPs) decreased smoking prevalence. Similar to findings from Langley et al.,^16^ this study also found that behavior change was linked to recent exposure. These findings suggest that it may be beneficial to sustain adequate levels of campaign exposure over the longer term. Another study found that increased exposure was associated with an increase in advertisement recall, and a reduction in average cigarette consumption and smoking prevalence.^33^ Again, none of these findings were specific to those with mental illness.

### Intentions to Quit

This review found inconclusive evidence on the effect of tobacco control MMCs on intentions to quit among those with mental illness. This is yet to be assessed in another review. A review of reviews by Stead et al suggests that tobacco control MMCs can have a positive effect on intentions to quit among general populations.^10^ However, in Stead et al.’s article, while the two included reviews had a low risk of bias, the studies that they included were of medium to low quality.^10^

### Perceived Effectiveness

Quantitative data from this review suggest that perceived effectiveness of tobacco control campaigns was greater among people with mental illness compared to those without.^24,30^ However, these findings are not conclusive as the data are not wholly comparable due to differences in how perceived effectiveness was measured. Additionally, both studies have a high risk of bias. Qualitative data from this review suggest that those with mental illness perceived tobacco control campaigns to be ineffective.^31^ However, these data should be regarded with caution as they were derived from one study with a high risk of bias, and which only included eight participants who all had SMI. Therefore, the results are not generalizable to the wider population of people with mental illness.

### Knowledge

Qualitative data from this review suggest that tobacco control MMCs can impart knowledge of the negative physical health impacts of tobacco use, among those with mental illness.^31^ However, findings also suggest that among this population there are perceptions that tobacco use has a positive impact on mental health. Similar findings were found in a Cochrane review by Bala et al.^14^ although this review focused on general populations. Bala et al. found some increase in knowledge about smoking and cardiovascular risk factors among adults following state-wide and community-based tobacco control MMCs.^14^ However, the studies included in this review were heterogenous and tended to have a high risk of bias. A review of reviews found mixed evidence of the effect of MMCs on knowledge of tobacco and smoking cessation helplines in general populations.^10^ Two reviews reported mixed results, two reported positive results and one reported negative results. All five reviews had a low risk of bias.

### Strengths and Limitations

To the authors’ knowledge, this is the first systematic review to assess the effect of tobacco control MMCs on smoking-related behavior among people with mental illness. One of the main strengths of this systematic review is the robust approach used to identify and synthesize relevant literature. This included a detailed eligibility criteria; a thorough search strategy; independent double screening, data extraction and risk of bias assessment processes; and a suitable synthesis given the heterogeneity between included studies. Furthermore, these processes were outlined a priori in the registered and published study protocol: https://osf.io/gda3y/.

The main limitation is that only eight eligible studies were identified and included. None of these studies assessed cost-effectiveness, so Objective 2 could not be completed. The included evidence also had limitations. First, there was considerable heterogeneity between studies especially in study design, length of follow-up, independent variables, and outcomes. Therefore, in most instances, data were not directly comparable. Second, all but one of the studies had a high risk of bias, and the remaining study had a moderate risk of bias. The synthesis could have been improved by giving more weight to the study that had a moderate risk of bias, although there is a lack of consensus about how this should be done within a narrative synthesis.^34^

### Implications and Future Research

Minimizing bias is a key challenge when designing studies to evaluate MMCs. As with this review, most evidence on MMC effectiveness is derived from observational studies in which bias is inherent. Although RCTs are less prone to bias and they can demonstrate causality, they may not provide evidence of real-world implementation. Therefore, careful consideration should be given to the design of future studies that evaluate MMCs in order to provide the best possible quality of evidence.

Due to the limited evidence, there are no recommendations for practice based on this review. Further research should examine the need for tobacco control MMC messages that address mental health. Such research should consider the needs of different mental health populations. For instance, those with general mental illness and those with SMI may respond differently to MMC messages, meaning a tailored approach may be warranted.

## Conclusions

Findings from this systematic review should be interpreted with caution as data are limited and are at a moderate to high risk of bias. There is evidence to suggest that tobacco control MMCs have limited impact on those with mental illness, though there is a potential influence on quitting behaviors if the campaign advertisement is specific to smoking and mental health. However, further research is needed.

## Supplementary Material

A Contributorship Form detailing each author’s specific involvement with this content, as well as any supplementary data, are available online at https://academic.oup.com/ntr.

ntac079_suppl_Supplementary_Material

ntac079_suppl_Supplementary_Taxonomy-form

## Data Availability

The protocol (PROSPERO registration number: CRD42021236066) and supporting documents are published on the OSF website: https://osf.io/gda3y/.
