# Supplementary material for: The Effect of Tobacco Control Mass Media Campaigns on Smoking-Related Behavior Among People With Mental Illness: A Systematic Literature Review
Source: Nicotine Tob Res. 2022 Mar 31;24(11):1695–704. doi: 10.1093/ntr/ntac079 (PMC9597075; doi:10.1093/ntr/ntac079)
Supplement: ntac079_suppl_Supplementary_Material [file ntac079_suppl_supplementary_material.docx]

**S1: Search terms and the search strategy**

*Medline, Embase and PsycInfo (30/03/2021)*: (tobacco or nicotine or cigar* or e-cig* or "electronic cigarette" or smok* or vap*).af. AND (((((((((((((((((((((((((((((((((((((((((((((((((((((((((((((("mass media" or multi-media) and campaign*) or media) and campaign*) or campaign*) and messag*) or health) and messag*) or health) and campaign*) or "social marketing") and campaign*) or "social marketing") and messag*) or broadcast*) and campaign*) or televis*) and campaign*) or TV) and campaign*) or film*) and campaign*) or radio) and campaign*) or advert*) and campaign*) or internet) and campaign*) or online) and campaign*) or email*) and campaign*) or website*) and campaign*) or digital*) and campaign*) or "social media") and campaign*) or Facebook) and campaign*) or Twitter) and campaign*) or Instagram) and campaign*) or TikTok) and campaign*) or phon*) and campaign*) or app*) and campaign*) or newspaper*) and campaign*) or magazine*) and campaign*) or leaflet*) and campaign*) or booklet*) and campaign*) or billboard*) and campaign*) or poster*) and campaign*).af. AND ((((((((((mental adj2 illness*) or "psychiatric illness*").af. or mental.mp.) adj2 health.af.) or mental.mp.) adj2 condition*.af.) or mental.mp.) adj2 disorder*.af.) or "psychiatric disorder*".af. or "psychiatric disease*".af. or anxi*.af. or depress*.af. or "obsessive compulsive disorder".af. or OCD.af. or "bipolar disorder".af. or "manic depress*".af. or schizophreni*.af. or psycho*.af. or paranoi*.af. or "personality disorder".af. or "attention deficit hyperactivity disorder".af. or ADHD.af. or "post traumatic stress disorder".af. or PTSD.af. or eating.mp.) adj1 disorder*.af.) or anorexi*.af. or bulimi*.af. or "binge eating disorder".af. or BED.af. or "panic attack*".af. or "panic disorder".af. or phobi*.af. (limit to English language and humans)

*Web of Science (30/03/2021)*: (tobacco or nicotine or cigar* or e-cig* or "electronic cigarette" or smok* or vap*) AND (((((((((((((((((((((((((((((((((((((((((((((((((((((((((((((((("mass media") or multi-media) and campaign*) or media) and campaign*) or campaign*) and messag*) or health) and messag*) or health) and campaign*) or "social marketing") and campaign*) or "social marketing") and messag*) or broadcast*) and campaign*) or televis*) and campaign*) or TV) and campaign*) or film*) and campaign*) or radio) and campaign*) or advert*) and campaign*) or internet) and campaign*) or online) and campaign*) or email*) and campaign*) or website*) and campaign*) or digital*) and campaign) or "social media") and campaign*) or Facebook) and campaign*) or Twitter) and campaign*) or Instagram) and campaign*) or TikTok) and campaign*) or phon*) and campaign*) or app*) and campaign*) or newspaper*) and campaign*) or magazine*) and campaign*) or leaflet*) and campaign*) or booklet*) and campaign*) or billboard*) and campaign*) or poster*) and campaign*) AND TS=(mental NEAR/2 illness*) OR TS=(“psychiatric illness*”) OR TS=(mental NEAR/2 health) OR TS=(mental NEAR/2 condition*) OR TS=(mental NEAR/2 disorder*) OR TS=(“psychiatric disorder*”) OR TS=(“psychiatric disease*”) OR TS=(anxi*) OR TS=(despress*) OR TS=(“obsessive compulsive disorder”) OR TS=(OCD) OR TS=(“bipolar disorder”) OR TS=(“manic depress*”) OR TS=(schizopreni*) OR TS=(psycho*) OR TS=(paranoi*) OR TS=(“personality disorder”) OR TS=(“attention deficit hyperactivity disorder”) OR TS=(ADHD) OR TS=(“post traumatic stress disorder”) OR TS=(PTSD) OR TS=(eating NEAR/1 disorder*) OR TS=(anorexi*) OR TS=(bulimi*) OR TS=(“binge eating disorder”) OR TS=(BED) OR TS=(“panic attack*”) OR TS=(“panic disorder”) OR TS=(phobi*) (limit to English language)

*CINAHL (30/03/2021)*: (tobacco or nicotine or cigar* or e-cig* or "electronic cigarette" or smok* or vap*) AND (((((((((((((((((((((((((((((((((((((((((((((((((((((((((((((("mass media" or multi-media) and campaign*) or media) and campaign*) or campaign*) and messag*) or health) and messag*) or health) and campaign*) or "social marketing") and campaign*) or "social marketing") and messag*) or broadcast*) and campaign*) or televis*) and campaign*) or TV) and campaign*) or film*) and campaign*) or radio) and campaign*) or advert*) and campaign*) or internet) and campaign*) or online) and campaign*) or email*) and campaign*) or website*) and campaign*) or digital*) and campaign*) or "social media") and campaign*) or Facebook) and campaign*) or Twitter) and campaign*) or Instagram) and campaign*) or TikTok) and campaign*) or phon*) and campaign*) or app*) and campaign*) or newspaper*) and campaign*) or magazine*) and campaign*) or leaflet*) and campaign*) or booklet*) and campaign*) or billboard*) and campaign*) or poster*) and campaign*) AND ((((((((((mental N2 illness*) or "psychiatric illness*") or mental) N2 health) or mental) N2 condition*) or mental) N2 disorder*) or "psychiatric disorder*" or "psychiatric disease*" or anxi* or depress* or "obsessive compulsive disorder" or OCD or "bipolar disorder" or "manic depress*" or schizophreni* or psycho* or paranoi* or "personality disorder" or "attention deficit hyperactivity disorder" or ADHD or "post traumatic stress disorder" or PTSD or eating) N1 disorder*) or anorexi* or bulimi* or "binge eating disorder" or BED or "panic attack*" or "panic disorder" or phobi* (limit to English language)

*Cochrane trials only and Cochrane reviews only (31/03/2021)*: tobacco OR nicotine OR cigar* OR e-cig* OR “electronic cigarette” OR smok* OR vap* AND “mass media” OR multi-media AND campaign* OR media AND campaign* OR campaign* AND messag* OR health AND messag* OR health AND campaign* OR “social marketing” AND campaign* OR “social marketing” AND messag* OR broadcast* AND campaign* OR televis* AND campaign* OR TV AND campaign* OR film* AND campaign* OR radio AND campaign* OR advert* AND campaign* OR internet AND campaign* OR online AND campaign* OR email* AND campaign* OR website* AND campaign* OR digital* AND campaign* OR “social media” AND campaign* OR Facebook AND campaign* OR Twitter AND campaign* OR Instagram AND campaign* OR TikTok AND campaign* OR phon* AND campaign* OR app* AND campaign* OR newspaper* AND campaign* OR magazine* AND campaign* OR leaflet* AND campaign* OR booklet* AND campaign* OR billboard* AND campaign* OR poster* AND campaign* AND mental NEAR/2 illness* OR “psychiatric illness*” OR mental NEAR/2 health OR mental NEAR/2 condition* OR mental NEAR/2 disorder* OR “psychiatric disorder*” OR “psychiatric disease*” OR anxi* OR depress* OR “obsessive compulsive disorder” OR OCD OR “bipolar disorder” OR “manic depress*” OR schizophreni* OR psycho* OR paranoi* OR “personality disorder” OR “attention deficit hyperactivity disorder” OR ADHD OR “post traumatic stress disorder” OR PTSD OR eating NEAR/1 disorder* OR anorexi* OR bulimi* OR “binge eating disorder” OR BED OR “panic attack*” OR “panic disorder” OR phobi*

Reference lists of included studies and relevant systematic reviews were also screened to retrieve further articles.

**S2: Data items**

Data extraction sheet headings:

- Author(s), year and title
- Country and setting
- Study design
- Participants and sample size
- Intervention/exposure (including dose and duration)
- Control/comparator
- Outcome(s)
- Measurement of outcome(s)
- Key findings
- Funder
- Target group
- Smoking definition
- Mental health definition/measurement
- Recruitment methods
- Data collection dates and methods
- Additional information

**S3: EPHPP tool risk of bias assessment independent reviewer ratings**

|  | **Selection bias** | | | **Study design** | | | **Confounders** | | **Blinding** | | **Data collection methods** | | | **Withdrawals and dropouts** | | | **Global rating** | |
| --- | --- | --- | --- | --- | --- | --- | --- | --- | --- | --- | --- | --- | --- | --- | --- | --- | --- | --- |
| Reviewer | PPH | BT | PPH | | BT | PPH | | BT | PPH | BT | | PPH | BT | | PPH | BT | PPH | BT |
| Davis 2017 | / | / | / | | / | + | | + | - | - | | - | - | | / | / | - | - |
| Davis 2018 | + | + | - | | / | + | | + | - | - | | - | - | | - | - | - | - |
| McAfee 2017 | - | - | + | | - | / | | + | - | - | | / | - | | ? | - | - | - |
| Neff 2016 | / | / | / | | / | + | | + | - | - | | / | - | | / | - | / | - |
| Nonnemaker 2014 | / | / | - | | - | + | | + | - | - | | - | - | | ? | ? | - | - |
| Prochaska 2018 | / | / | / | | / | + | | + | - | - | | / | - | | - | - | - | - |
| Thornton 2011 | - | - | - | | - | - | | - | - | - | | - | - | | ? | ? | - | - |
| Thornton 2013 | - | / | - | | - | / | | / | - | - | | + | + | | ? | ? | - | - |

+: low risk of bias; /: moderate risk of bias; -: high risk of bias; ?: not applicable

**S4: EPHPP tool risk of bias assessment consensus ratings**

|  | **Selection bias** | | **Study design** | | **Confounders** | **Blinding** | **Data collection methods** | | **Withdrawals and dropouts** | | **Global rating** |
| --- | --- | --- | --- | --- | --- | --- | --- | --- | --- | --- | --- |
|  |  | |  | |  |  |  | |  | |  |
| Davis 2017 | / | / | | + | | - | | - | | / | - |
| Davis 2018 | + | - | | + | | - | | - | | - | - |
| McAfee 2017 | - | + | | + | | - | | / | | ? | - |
| Neff 2016 | / | / | | + | | - | | / | | / | / |
| Nonnemaker 2014 | / | - | | + | | - | | - | | ? | - |
| Prochaska 2018 | / | / | | + | | - | | / | | - | - |
| Thornton 2011 | - | - | | - | | - | | - | | ? | - |
| Thornton 2013 | - | - | | / | | - | | + | | ? | - |

+: low risk of bias; /: moderate risk of bias; -: high risk of bias; ?: not applicable

**S5: CASP Checklist appraisal tool independent reviewer ratings**

|  | **Statement of aims** | | | **Appropriate methodology** | | | **Appropriate design** | | **Appropriate recruitment strategy** | | **Appropriate data collection** | | | **Consideration of researcher & participant relationship** | | | **Consideration of ethical issues** | | **Rigorous data analysis** | | **Statement of findings** | | |
| --- | --- | --- | --- | --- | --- | --- | --- | --- | --- | --- | --- | --- | --- | --- | --- | --- | --- | --- | --- | --- | --- | --- | --- |
| Reviewer | PPH | BT | PPH | | BT | PPH | | BT | PPH | BT | | PPH | BT | | PPH | BT | PPH | BT | PPH | BT | PPH | BT |  |
| Thornton 2011 | + | + | + | | + | + | | + | ? | + | | ? | + | | - | ? | - | ? | ? | + | + | + |  |

+: yes; -: no; ?: can’t tell

**S6: CASP Checklist appraisal tool consensus ratings**

|  | **Statement of aims** | | **Appropriate methodology** | | **Appropriate design** | **Appropriate recruitment strategy** | **Appropriate data collection** | | **Consideration of researcher & participant relationship** | | **Consideration of ethical issues** | **Rigorous data analysis** | **Statement of findings** | |
| --- | --- | --- | --- | --- | --- | --- | --- | --- | --- | --- | --- | --- | --- | --- |
| Thornton 2011 | + | + | | + | | ? | | ? | | ? | ? | + | + |  |

+: yes; -: no; ?: can’t tell

**S7: Reasons for exclusion (based on full-text screen)**

|  | Author, year | Reason for exclusion |
| --- | --- | --- |
| 1 | Baggett et al, 2019 | Participants were not exposed to mass media campaign health messages |
| 2 | Bowden et al, 2011 | Participants were not exposed to mass media campaign health messages & outcomes were not relevant |
| 3 | Campbell et al, 2016 | Psychiatric comorbidity was not assessed |
| 4 | Coletti et al, 2015 | Participants were not exposed to mass media campaign health messages |
| 5 | Filia et al, 2014a | Outcomes were not relevant |
| 6 | Filia et al, 2014b | Participants were not exposed to mass media campaign health messages |
| 7 | Kruse et al, 2019 | Participants did not have a history of mental ill-health & were not exposed to mass media campaign health messages |
| 8 | Kruse et al, 2020 | Participants did not have a history of mental ill-health & were not exposed to mass media campaign health messages |
| 9 | Latha et al, 2020 | Outcomes were not relevant |
| 10 | Rodevand et al, 2019 | Participants were not exposed to mass media campaign health messages |
| 11 | Sharma et al, 2016 | There were no human participants (this was an analysis of the quality & quantity of quit-smoking YouTube videos) & outcomes were not relevant |
| 12 | Sharma-Kumar et al, 2018 | Participants were not exposed to mass media campaign health messages |

**S8: Results from the included studies organised by reported outcomes and length of follow-up***

| Author, year | **Quit attempts (QA, ≥1 attempt lasting ≥24 hours)** | **Ad recall/ad exposure** | **Intentions to quit (ITQ)** | **Perceived effectiveness (PE)** | **Risk of bias** |
| --- | --- | --- | --- | --- | --- |
| Neff et al, 2016^26^ | **Past 3 months**  **Pre-post change in QAs**  *With MH condition (n=2,536)*  OR 0.98, 95% CI: 0.77-1.26, P=0.91  *Without MH condition* *(n=5,199)*  **OR 1.24, 95% CI: 1.04-1.49, P=0.02** | N/A | **Next 30 days**  **Pre-post change in ITQ**  *With MH condition (n=2,349)*  OR 1.09, 95% CI: 0.78-1.52 , P=0.61  *Without MH condition (n=4,783)*  OR 1.41, 95% CI: 0.96-2.05, P=0.08  **Next 6 months**  **Pre-post change in ITQ**  *With MH condition (n=2,349)*  OR 1.10, 95% CI: 0.83-1.46 , P=0.51  *Without MH condition (n=4,783)*  **OR 1.38, 95% CI: 1.06-1.80, P=0.02** | N/A | Mod |
| Davis et al, 2017^24^ | **Past 3 months**  **Association between baseline PE & QA**  *With (n=2,214) vs without (n is missing) MH condition*  OR 1.06, 95% CI: 0.86-1.32 | N/A | N/A | **Level of PE**  *With (n=2,214) vs without (n is missing) MH condition*  **b=0.12, 95% CI: 0.07-0.17, p<0.01** | High |
| Davis et al, 2018^25^ | **Past 3 months**  **Association between GRPs & QAs**  *Overall sample (associations by MH status not reported; n=22,965 observations)*  **AOR 1.23 (1.11-1.36), p<0.001**  **Interaction between GRPs & MH status for QAs**  No sig interaction | N/A | **Next 30 days**  **Association between GRPs & ITQ**  *Overall sample (associations by MH status not reported; n=22,271 observations)*  **AOR 1.17 (1.02-1.36), p=0.030**  **Interaction between GRPs & MH status for ITQ**  No sig interaction | N/A | High |
| Prochaska et al, 2018^27^ | **Past 6 months**  **At follow up**  *With vs without MH condition*  **51.1.% vs 43.7%, p<0.05**  **Association between increased exposure to mental health specific ad & QA**  *With MH condition (n=772)*  **AOR 1.25, 95% CI: 1.03-1.52, p<0.05**  *Without MH condition (n=1804)*  AOR 0.97, 95% CI: 0.83-1.14, p=0.737  **Association between increased exposure to non-mental health specific ads & QA**  *With MH condition (n=775)*  AOR 1.09, 95% CI: 0.88-1.35, p=0.43  *Without MH condition (n=1805)*  **AOR 1.19, 95% CI: 1.02-1.40, p<0.05** | **Reported seeing ≥1 Tips ad**  *With vs without MH condition* 84% vs 79.8%  **Reported seeing mental health specific ad**  *With vs without MH condition*  53.4% vs 48.3%  **Reported seeing ≥1 non mental health specific ad**  *With vs without MH condition*  82.5% vs 78.7% | **Next 30 days**  **At follow up**  *With vs without MH condition*  12.2% vs 10.6%, p=0.530  **Association between increased exposure to mental health specific ad & ITQ**  *With MH condition (n=694)*  **AOR 1.40, 95% CI: 1.04-1.90, p<0.05**  *Without MH condition (n=1670)*  AOR 1.17, 95% CI: 0.93-1.46, p=0.174  **Association between increased exposure to non-mental health specific ads & ITQ**  *With MH condition (n=695)*  AOR 1.22, 95% CI: 0.91-1.64, p=0.188  *Without MH condition (n=1671)*  AOR 1.14, 95% CI: 0.92-1.42, p=0.238 | N/A | High |
| McAfee et al, 2017^28^ | **Since campaign launch (up to past 6 months & 27 days)**  **QAs in standard dose market (761 National GRPS)**  *With vs without MH condition*  **42.5% vs 32.0%; p<0.01**  **QAs in higher dose market (758 National GRPS + 1,724 local GRPs) vs standard dose market (761 National GRPs)**  *With MH condition*  39.5% vs 42.5%, p=0.797; AOR 0.93, p=0.689  *Without MH condition*  **38.5% vs 32.0%, p=0.008; AOR 1.37, p=0.003** | N/A | N/A | N/A | High |
| Nonnemaker et al, 2014^29^ | **Past 12 months**  **At follow up**  *With vs without MH condition*  57.7% vs 53.1%  **Association between confirmed ad recall & QA**  *With MH condition (n=1,800)*  OR 1.07, 95% CI: 0.75-1.53  *Without MH condition (n=6,021)*  **OR 1.46, 95% CI: 1.17-1.82, p<0.001**  **Association between confirmed recall of graphic/emotional ads & QA**  *With MH condition (n=1,772)*  OR 1.12, 95% CI: 0.77-1.64  *Without MH condition (n=5,919)*  **OR 1.54, 95% CI: 1.21-1.95, p<0.001**  **Association between past year GRPs & QA**  *With MH condition (n=2,019)*  OR 1.13, 95% CI: 0.87-1.46  *Without MH condition (n=6,758)*  **OR 1.33, 95% CI: 1.16-1.53, p<0.001**  **Association between past year graphic/emotional ad GRPs & QA**  *With MH condition (n=2,019)*  OR 1.27, 95% CI: 0.89-1.82  *Without MH condition (n=6,758)*  **OR 1.49, 95% CI: 1.24-1.79, p<0.001**  **Association between past year comparison ad GRPs & QA**  *With MH condition (n=2,019)*  OR 0.9, 95% CI: 0.62-1.30  *Without MH condition (n=6,758)*  OR 1.1, 95% CI: 0.89-1.36 | N/A | N/A | N/A | High |
| Thornton et al, 2013^30^ | N/A | **Recall ≥1 tobacco campaign**  *With vs without psychotic disorder*  96.3% vs 96.9% | N/A | **Perceived campaigns regarding tobacco use to be effective**  *With vs without psychotic disorder*  **55.8% vs 44.7%, p<0.05** | High |
| Thornton et al, 2011^31^ Quantitative data  Qualitative data | N/A  N/A | (All participants had a psychotic disorder)  **Had seen, read or heard a public health campaign regarding tobacco**  93.2% (n=82/88)  **Exposure of current & past smokers, & non-smokers**  No sig differences (missing data)  N/A | N/A  N/A | N/A  See Table 2 | High |

Data in bold type indicates significance.

Ad(s): advertisement(s); GRPs: gross rating points; ITQ: intention(s) to quit; MH: mental health; mod: moderate; PE: perceived effectiveness; QA: quit attempt(s); sig: significant

*See Table 2 for results for the outcome “knowledge”
